# Lateral retroperitoneal approach for the treatment of a recumbent silkworm-like paravertebral schwannoma in the lumbar spine: a case report

**DOI:** 10.3389/fonc.2025.1544175

**Published:** 2025-03-18

**Authors:** Lanming Su, Wei Liu, Yingying Zhang, Feng Li, Yuanqin Liu, Ziyu Wang

**Affiliations:** ^1^ The First Affiliated Hospital of Shandong First Medical University and Shandong Provincial Qianfoshan Hospital, Shandong First Medical University& Shandong Academy of Medical Sciences, Jinan, China; ^2^ Department of Neurology, The First Affiliated Hospital of Shandong First Medical University and Shandong Provincial Qianfoshan Hospital, Jinan, China; ^3^ Department of Neurosurgery, The First Affiliated Hospital of Shandong First Medical University and Shandong Provincial Qianfoshan Hospital, Jinan, China; ^4^ School of Pharmacy, Shandong Second Medical University, Weifang, China

**Keywords:** lateral retroperitoneal approach, recumbent silkworm-like, schwannoma, lumbar, anterolateral retroperitoneal approach

## Abstract

**Objective:**

This case report describes the surgical management of a recumbent silkworm-like paravertebral schwannoma in the lumbar spine resected via the lateral retroperitoneal approach.

**Methods:**

A lumbar paravertebral schwannoma resembling a “recumbent silkworm” was excised using the lateral retroperitoneal approach. Intraoperative measures included electrophysiological monitoring and insertion of a ureteral D-J stent to protect critical surrounding anatomical structures. Complete tumor resection was achieved without intraoperative complications. Follow-up demonstrated no tumor recurrence or postoperative complications.

**Results:**

The duration of surgery was 310 min, with an estimated blood loss of 50mL. The tumor resection rate was 100%. The patient was discharged 11 days after hospitalization. Postoperative histopathological examination confirmed the diagnosis of schwannoma. There were no postoperative complications. The patient ADL (Activities of Daily Living score did not decline compared with the preoperative levels. After one year of follow-up, no signs of tumor recurrence were observed.

**Conclusion:**

Paravertebral tumors located in the anterior and lateral aspects of the lumbar spine pose significant surgical challenges because of their complex position and morphology. Complete excision of such tumors via the lateral retroperitoneal approach is an optimal treatment strategy.

## Introduction

Schwannomas are rare tumors originating from Schwann cells of the peripheral nerve sheath (1) and typically present as isolated or multiple lesions in the head, neck, and extremities (2). Retroperitoneal schwannomas are sporadic, constituting only 1–3% of all schwannomas (1), and are most commonly observed in patients aged 40–60 years (3). These tumors are generally benign unless associated with neurofibromatosis type 1 (von Recklinghausen’s disease) or radiation exposure (10–20%) (4, 5). Based on imaging characteristics, Eden classified schwannomas into four types in 1941 (6). Type I involves intradural and extradural components within the spinal canal; Type II involves both the spinal canal and the paravertebral space; Type III affects the paravertebral area without intradural involvement; and Type IV primarily involves the intervertebral foramen and paravertebral regions. Pure paravertebral lesions are exceedingly rare (7).

Complete surgical resection is the preferred treatment and is associated with favorable outcomes (8). Traditional approaches for surgical resection include posterior midline, paraspinal intermuscular, and anterior laparoscopic approaches, although these techniques are often limited by lengthy operative times, technical difficulty, increased trauma, significant blood loss, and a higher risk of complications (9). Minimally invasive techniques that reduce tissue damage shorten hospital stays, and decrease blood loss have gained popularity in recent years. The oblique lumbar interbody fusion (OLIF) technique allows entry into the retroperitoneal space through a natural muscle-vascular gap (10). The feasibility and safety of the lateral retroperitoneal approach have been confirmed by anatomical and MRI studies (11). However, reports on the application of the posterolateral retroperitoneal approach for managing paraspinal tumors are rarely documented in existing literature. In this case, given the unique morphology of the tumor, our team opted for the lateral retroperitoneal approach for surgical excision of the recumbent silkworm-like paravertebral tumor. The details of this case are presented below:

## Case report

### Medical history

**Figure 1:**
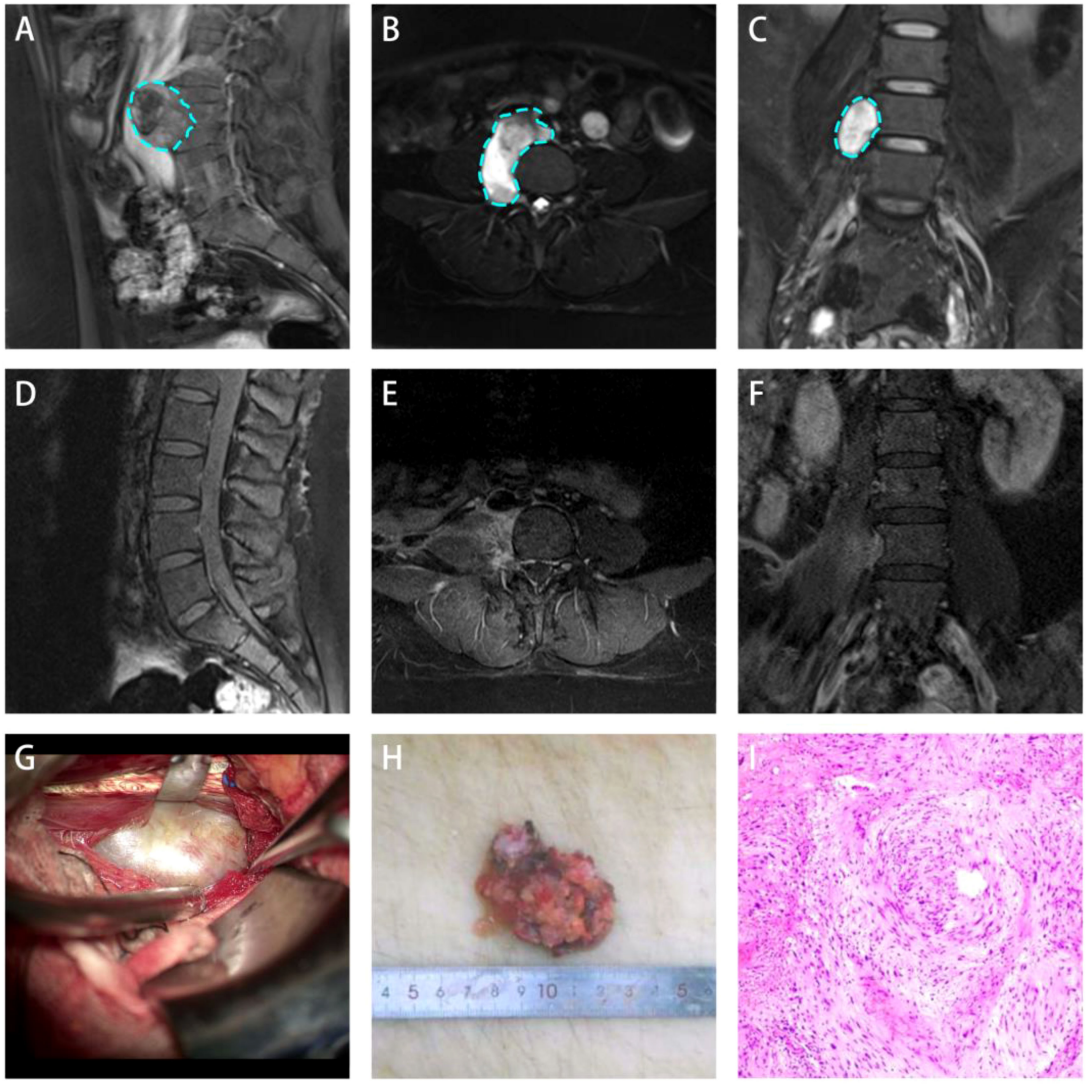
Lateral retroperitoneal approach for treating lumbar spine anterolateral schwannoma. **(A-C)** Preoperative images showing a recumbent silkworm-like high-signal area in the anterolateral retroperitoneal region adjacent to the lumbar vertebrae. **(D-F)** One-month postoperative follow-up images showed no signs of tumor recurrence. **(G)** Intraoperative microscopic view of the tumor capsule exposure. **(H)** Tumor measures approximately 5.5 cm in diameter. **(I)** Postoperative histopathological examination (H&E staining) confirmed the diagnosis of schwannoma.

A 22-year-old female presented with a 10-day history of dull abdominal pain of an unknown etiology. The pain did not improve despite conservative management, and no associated symptoms, such as nausea, vomiting, diarrhea, jaundice, or fever, were reported. The patient had no significant medical history, including chronic diseases or tumors, and her menstrual cycle was regular. On physical examination, there was no paravertebral tenderness, spinal mobility was normal, muscle strength in both lower extremities was 5/5, muscle tone was normal, and pathological reflexes were absent. Lumbar MRI demonstrated a round lesion adjacent to the L4-5 vertebral bodies on the right side (**Figures 1A–C**). The lesion demonstrated homogeneous long T1 and T2 signals and extended from the anterior right aspect of the vertebral body to the right intervertebral foramen through the psoas muscle (**Figure 1B**). Enhanced MRI demonstrated markedly delayed enhancement with non-enhancing areas within the lesion.

### Surgical procedure

After completing routine preoperative assessments, no contraindications for surgery were identified. Based on imaging findings, tumor resection was performed using the lateral retroperitoneal approach. Following anesthesia induction, the patient was placed in the lithotomy position. A urologist inserted a ureteral D-J stent to protect the ureter during the surgery. The patient was placed in the left lateral decubitus position. A 10 cm incision was made in the right lower abdomen, and the surgical area was prepared. The skin, subcutaneous tissue, external oblique muscle, internal oblique muscle, and transverse abdominal muscle were dissected layer by layer. On deep dissection, the tumor was identified as being anterior and inferior to the psoas muscle (**Figure 1G**). The tumor was firm, grayish-white in color, had well-defined borders and was located adjacent to the right ureter and inferior vena cava. The tumor capsule was then incised, and piecemeal tumor resection was performed to decompress the tumor. Careful dissection was continued along the tumor capsule, and the root of the tumor was found in the deep psoas muscle, which is closely related to several nervous structures. The tumor was excised in parts (**Figure 1H**). After hemostasis was ensured, the muscle was restored, a drainage tube was placed, and the wound was closed in layers using absorbable sutures. The tumor was sent for pathological analysis. The surgery was completed successfully with an estimated blood loss of 50mL and no need for blood transfusion.

### Pathology and follow-up

Postoperative pathological and immunohistochemical examinations demonstrated that the tumor surface was partially smooth and partially rough. The cut surface appeared gray–white to gray–red with a soft texture. The pathological diagnosis confirmed schwannoma (**Figure 1I**) with localized sclerosis. Immunohistochemistry demonstrated S-100 (+), SOX-10 (+), EMA (-), and Ki-67 (approximately 5%). Aside from the mild incision pain, the patient reported no significant discomfort. A physical examination demonstrated no obvious positive signs. The patient was discharged after one week of comprehensive treatment, including hemostasis and swelling reduction. One month after surgery, lumbar spine MRI showed complete tumor resection (**Figures 1D–F**). The ADL score showed no significant decline compared to the preoperative levels. At the one-year follow-up, no significant tumor recurrence was observed.

## Discussion

The retroperitoneal space offers considerable flexibility owing to its large volume, and retroperitoneal schwannomas typically grow slowly. Therefore, symptoms are often minimal or appear late (3, 12). The clinical presentation is primarily determined by the size and location of the tumor and tends to be highly nonspecific (3). Abdominal pain and neurological deficits are the most common symptoms (13), but there are reports of atypical symptoms such as headaches, secondary hypertension, and renal colic with hematuria (14). Paravertebral schwannomas respond poorly to both radiation therapy and chemotherapy, making complete surgical resection the best therapeutic option. The choice of surgical approach is dictated by the size, location, and proximity of the tumor to surrounding structures (7). Traditional surgical approaches include the posterior and anterior transabdominal approaches.

The posterior approach is widely used for the resection of paravertebral tumors and includes both the midline posterior approach and the Wiltse paraspinal intermuscular approach. The posterior approach has the advantage of avoiding major blood vessels and nerves located anterior to the vertebral body, which improves surgical safety. The classic midline posterior approach involves the detachment of the paravertebral muscles and the removal of the lamina and facet joints on the affected side to access the tumor. After tumor resection, fusion of the affected vertebrae is often required to maintain spinal stability (15). Furthermore, for large paravertebral tumors, the midline posterior approach may necessitate extensive removal of the lamina to achieve adequate exposure (16). Christopher P. Wang et al. (17) reported the successful use of the Wiltse paraspinal approach to treat two cases of paravertebral tumors and suggested that this approach is ideal for large paravertebral schwannomas. It allows complete tumor resection with potential nerve preservation while maintaining spinal stability. However, In this case, no posterior approach could have adequately exposed the lesion.

The transabdominal anterior approach is another option for the resection of paravertebral tumors. This approach avoids the need for soft tissue detachment and bone removal from the paraspinal region, thereby reducing the risk of postoperative spinal instability (18). Some reports suggest that the anterior approach may reduce blood loss, alleviate postoperative pain, and shorten the hospital stay (19). However, the anterior approach carries significant risks, such as bowel obstruction due to excessive retraction of the intestines and psoas muscle injury from overextension (20). Additionally, if the tumor is located near the abdominal aorta or inferior vena cava, the risks associated with the procedure increase dramatically (21).

In this case, the unique growth pattern of the tumor posed a significant challenge. A thorough review of the literature demonstrated no reports of paravertebral tumors with similar morphology. First, it was a rare extraforaminal schwannoma, and second, its shape was not round but rather resembled a “ recumbent silkworm,” extending along the anterior and lateral aspects of the vertebral body and reaching the midline anteriorly. Traditional posterior approaches cannot adequately expose tumors located on the anterior aspect of the vertebral body. Moreover, the tumor was situated adjacent to the common iliac vessels, increasing the risk of the anterior approach. Additionally, the procedure itself carries a high risk of postoperative bowel obstruction owing to bowel retraction injuries. Given these considerations, we chose the lateral retroperitoneal approach and successfully resected the tumor. Lei Zhang et al. (22) reported the use of the lateral retroperitoneal approach in six cases of paravertebral tumors. However, the tumors in that series were all located laterally and exhibited more common shapes, making surgery relatively simple.

The lateral retroperitoneal approach allows direct access to the tumor through natural anatomical gaps between muscles, avoiding the destruction of vertebral structures and paraspinal soft tissues seen with the posterior approach, as well as the risks of bowel obstruction and significant blood loss associated with the anterior approach. Additionally, this approach provides direct exposure to tumors located on the anterolateral aspect of the vertebral body, which is crucial for complete resection. Furthermore, the incision size in the lateral retroperitoneal approach is smaller than that in both the transabdominal and posterior intermuscular approaches, facilitating faster postoperative recovery and alignment with the principles of Enhanced Recovery After Surgery (ERAS). Multidisciplinary collaboration, including ureteral D-J stent placement, intraoperative electrophysiological monitoring, and appropriate use of surgical instruments, can minimize the risk of injury to the ureter, sympathetic nerves, and lumbar nerve plexus.

## Conclusion

The lateral retroperitoneal approach for the treatment of anterolateral lumbar paravertebral tumors is considered the optimal choice, as it provides the best tumor exposure while protecting surrounding structures, significantly reducing the risk of intraoperative injury.

## Data Availability

The original contributions presented in the study are included in the article/supplementary material. Further inquiries can be directed to the corresponding authors.
